# Suspected Paramalignant Pleural Effusion as the Primary Presentation of Disseminated Cholangiocarcinoma in a Cat

**DOI:** 10.3390/vetsci13080834

**Published:** 2026-08-20

**Authors:** Kanoklada Koomgun, Wuttikij Ruangpoonga, Piraya Jaiyangyeun, Nattawipa Suwannasaeng, Chaiyan Kasorndorkbua, Naris Thengchaisri, Panpicha Sattasathuchana

**Affiliations:** 1 Department of Companion Animal Clinical Sciences, Faculty of Veterinary Medicine, Kasetsart University, Bangkok 10900, Thailand; kanoklada.k@ku.th (K.K.); fvetnrt@ku.ac.th (N.T.); 2 Veterinary Diagnostic Laboratory, Kasetsart University Veterinary Teaching Hospital, Kasetsart University, Bangkok 10900, Thailand; wuttikij.ru@ku.th; 3 Kasetsart Veterinary Imaging and Radiotherapy Center, Kasetsart University Veterinary Teaching Hospital, Kasetsart University, Bangkok 10900, Thailand; piraya.ja@ku.th (P.J.); nattawipa.su@ku.th (N.S.); 4 Department of Pathology, Faculty of Veterinary Medicine, Kasetsart University, Bangkok 10900, Thailand; chaiyan.k@ku.th

**Keywords:** bile duct carcinoma, cat, cholangiocarcinoma, pleural effusion

## Abstract

A 12-year-old neutered male domestic shorthair cat presented with recurrent pleural effusion following repeated thoracentesis. Initial analysis of the pleural effusion did not reveal neoplastic cells. Thoracic imaging demonstrated pleural effusion without identifiable nodular lesions. However, abdominal computed tomography and histopathological examination of an ultrasound-guided percutaneous core liver biopsy identified a large hepatic mass, confirmed to be cholangiocarcinoma, with suspected metastases to the spleen. After exclusion of other potential causes, the effusion was suspected to be a paramalignant pleural effusion. Despite palliative management, the disease progressed, and the cat was euthanized 98 days after definitive diagnosis. Postmortem examination revealed widespread metastatic cholangiocarcinoma involving the lungs, spleen, and intercostal muscles. No gross mass lesions were identified in the pulmonary parenchyma. Histological results demonstrated neoplastic cell infiltration within both the pulmonary lymphatic and blood vessels, a finding consistent with lymphovascular spread, which can contribute to the development of pleural effusion. This case show that while the characteristics of neoplasia-associated pleural fluid may vary according to disease stage, cholangiocarcinoma-associated pleural effusion may occur in the absence of cytologic evidence of malignancy, pulmonary nodules on thoracic imaging, or typical hepatobiliary signs.

## Simple Summary

We describe a case of a 12-year-old neutered male domestic shorthair cat presented with recurrent pleural effusion and dyspnea. Initial pleural fluid cytology did not identify neoplastic cells, and thoracic imaging did not reveal pulmonary nodules. However, abdominal imaging revealed a large hepatic mass, and a liver biopsy confirmed the presence of cholangiocarcinoma, a rare and aggressive tumor in cats. After exclusion of other potential causes, the effusion was suspected to be paramalignant pleural effusion. Over time, malignant cells were identified, indicating that exfoliated neoplastic cells may appear later in repeated sampling despite metastatic disease elsewhere. The cat was ultimately humanely euthanized because of progressive neoplastic disease. A necropsy confirmed that there were widespread metastases to the spleen, lungs, and intercostal muscles. Histology showed neoplastic cell infiltration within pulmonary lymphatic and blood vessels, a finding consistent with lymphovascular spread, which may have contributed to the accumulation of pleural effusion. This case highlights the importance of considering extrathoracic malignancy in cats with recurrent pleural effusion, as cytologic findings may be inconclusive during the early stages of disease. Therefore, serial pleural fluid analysis is warranted in cats with recurrent pleural effusion, as the absence of discrete pulmonary nodules on imaging and negative pleural fluid cytology do not exclude extrathoracic malignancy.

## 1. Introduction and Clinical Significance

Pleural effusion is the abnormal accumulation of fluid within the pleural cavity. It is a nonspecific finding that can result from a variety of disease processes and is most commonly associated with thoracic disease in cats [1]. Physiologically, pleural fluid is removed through pressure gradients across the visceral pleura, lymphatic drainage via stomata in the parietal pleura, and transport mechanisms by the pleural mesothelial cells. Dysfunction of this fluid turnover can result in pleural effusion [2,3]. Clinical signs are usually related to fluid accumulation and commonly include difficulty breathing (45.3%), anorexia (20.0%), and lethargy (10.8%) [1,3].

Approximately 26% of pleural effusion cases in cats are associated with neoplasia [1]. Fluid analysis plays a crucial role in identifying the underlying cause, particularly in cases of neoplasia, wherein exfoliated neoplastic cells may be detected. However, neoplasia-associated pleural effusions can also be paramalignant [4,5,6]. Paramalignant effusions, a term widely used in human medicine, is defined as effusions associated with malignancy in the absence of direct neoplastic infiltration or detectable malignant cells in the fluid [7,8] and may delay diagnoses. The characteristics of neoplasia-associated effusion may vary and change over time as tumor infiltration progresses. Repeated pleural fluid analysis and cytologic evaluation may therefore improve diagnostic yields [4,7]. Primary pulmonary neoplasia is relatively rare in cats, whereas pulmonary metastases, particularly adenocarcinoma, are more common [9]. Pulmonary metastasis of carcinoma can occur via hematogenous, lymphangitic, or aerogenous routes [6,10].

Cholangiocarcinoma (bile duct carcinoma) is an uncommon neoplasm but represents the most common malignant non-hematopoietic hepatic tumor in cats [11,12,13,14,15]. It exhibits aggressive biological behavior with high rates of local invasion and 67–80% diffuse intraperitoneal metastasis to the regional lymph nodes, lungs, spleen, and other sites [14,15]. Reported cases typically presented with clinical signs related to the mass effect of the tumor, which are usually nonspecific and include lethargy, anorexia, weight loss, jaundice, and abdominal distension due to the mass or ascites. Compared with peritoneal effusion, pleural effusion has rarely been described in relation to feline cholangiocarcinoma [11,12,16,17,18,19,20,21]. Tumor-associated ascites, being closer to the primary lesion, is more commonly correlated with increased vascular permeability and inflammation or greater portal venous pressure caused by hepatic infiltration [3]. To the best of our knowledge, no reports have described cats with cholangiocarcinoma-associated pleural effusion in the absence of ascites and detectable pulmonary nodular lesions.

In this report, we describe the diagnostic challenges associated with an atypical presentation of feline cholangiocarcinoma, in which dyspnea due to suspected recurrent paramalignant pleural effusion was the predominant clinical finding rather than typical hepatobiliary abnormalities. By reporting this case, we aim to raise awareness of how recurrent pleural effusion serves as a potential manifestation of extrathoracic neoplasia in cats, even in the absence of detectable cytologic evidence of malignancy or pulmonary nodules on imaging.

## 2. Case Presentation

A 12-year-old neutered male domestic shorthair cat (with a body weight of 4.2 kg at the primary clinic) presented with recurrent pleural effusion. The cat was initially evaluated at a primary clinic following a 3-day history of respiratory distress with pleural effusion that rapidly reaccumulated within 1–3 days after repeated thoracenteses over a 30-day period before referral. However, the pleural fluid analysis conducted at the primary clinic was not included in the referral records. The cat was referred to the Kasetsart University Veterinary Teaching Hospital, Bangkhen Campus, for further diagnosis to determine the origin of the pleural effusion.

At the initial visit to the referral hospital, the cat presented with dyspnea and had a body condition score of 3/9 and a body weight of 3.9 kg. Hematology and blood chemistry evaluations were conducted using an automated hematology analyzer (Sysmex XN-1000^TM^ Hematology Analyzer, Sysmex, Lincolnshire, IL, USA) and an automatic chemistry analyzer (IL ILab 650 Chemistry System, Diamond Diagnostics, Holliston, MA, USA), respectively. Preprandial bile acid and ammonia were tested using a Catalyst One Chemistry Analyzer (IDEXX Laboratories, Inc., Westbrook, ME, USA), and the coagulation profiles were evaluated using an automated coagulation analyzer (CA-600 Series, Sysmex Corporation, Kobe, Japan). The cat exhibited normocytic, normochromic, nonregenerative anemia (Table 1). His serum biochemical and coagulation profiles were within normal limits, although mild hyperglycemia was noted (Table 2).

**Figure 1:**
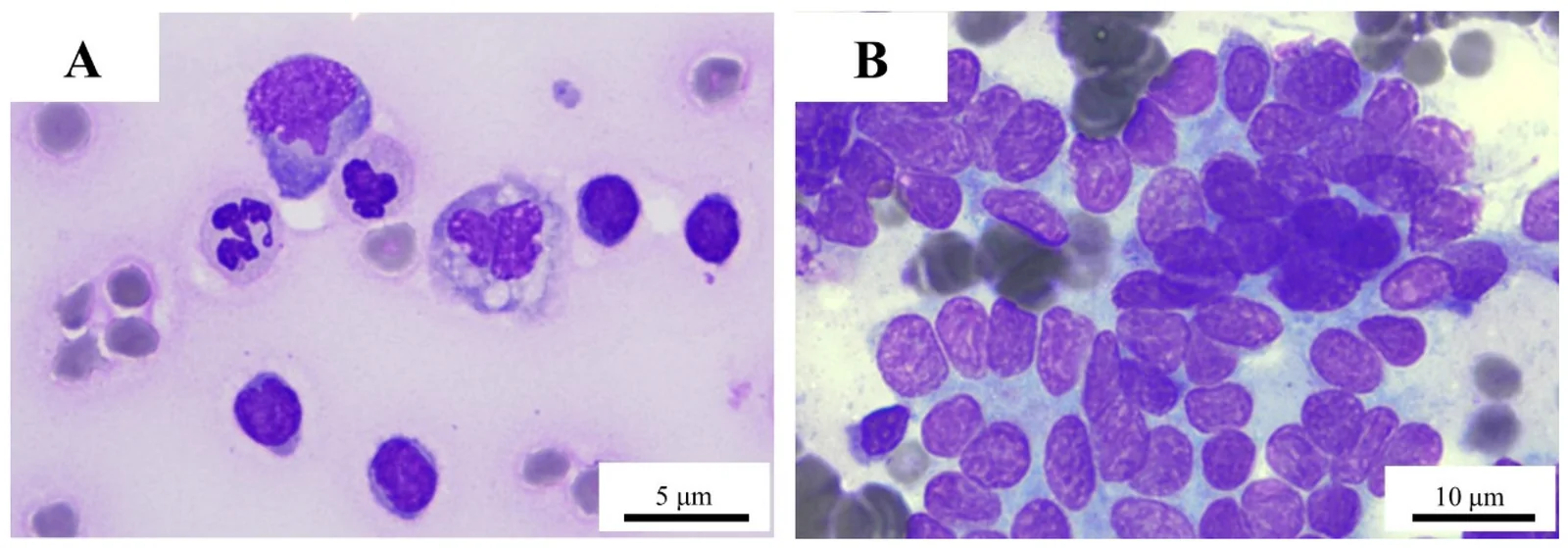
Cytological comparison of pleural effusion and hepatic mass aspirates from the affected cat. (**A**) A Wright–Giemsa-stained pleural-fluid smear, examined using a 100× oil-immersion objective, showed numerous small lymphocytes, a moderate quantity of nondegenerate neutrophils, and a few foamy macrophages. (**B**) Wright–Giemsa-stained fine-needle aspirate of the hepatic mass examined using a 100× oil-immersion objective, showed cohesive neoplastic epithelial cells arranged in acinar and palisading patterns. The cells had oval nuclei with one to three prominent nucleoli, anisokaryosis, and basophilic cytoplasm with occasional vacuoles.

Thoracic radiography showed pleural effusion with a diffuse interstitial pattern, while the diaphragm remained intact. The effusion was drained and repeatedly evaluated on two occasions at the initial visit and 10 days later. The fluid was light serosanguineous and classified as a modified transudate on the basis of a cytological analysis (specific gravity, 1.026 and 1.030; total nucleated cell count [TNCC], 1923/µL and 2940/µL; total protein, 35 and 41 g/L; and fluid-to-serum total protein ratio,0.50 and 0.63; respectively). Pleural fluid samples were processed via cytocentrifugation before cytological evaluation. The fluid was characterized by predominantly small monomorphic lymphocytes, moderate quantities of nondegenerate neutrophils, and a few foamy macrophages (Figure 1A). No neoplastic cells were identified, and both aerobic and anaerobic bacterial cultures were negative. Post-thoracentesis ultrasonography revealed mild residual pleural effusion without evidence of a lung mass, while echocardiography indicated normal cardiac function. Abdominal ultrasonography (LOGIQ E10, GE, Fairfield, CT, USA) with a 13 MHz broadband linear transducer revealed marked hepatomegaly with a large, irregular heterogeneous mass (4.9 × 4.9 cm) adjacent to the gallbladder. A heterogeneous splenic nodule (1.2 × 0.77 cm) and multiple hypoechoic pancreatic nodular lesions throughout the parenchyma were also identified. No evidence of portal hypertension was noted judging by the normal portal vein parameters (velocity 16 cm/s; portal-vein-to-aorta ratio, 0.9) and absence of peritoneal effusion.

**Figure 2:**
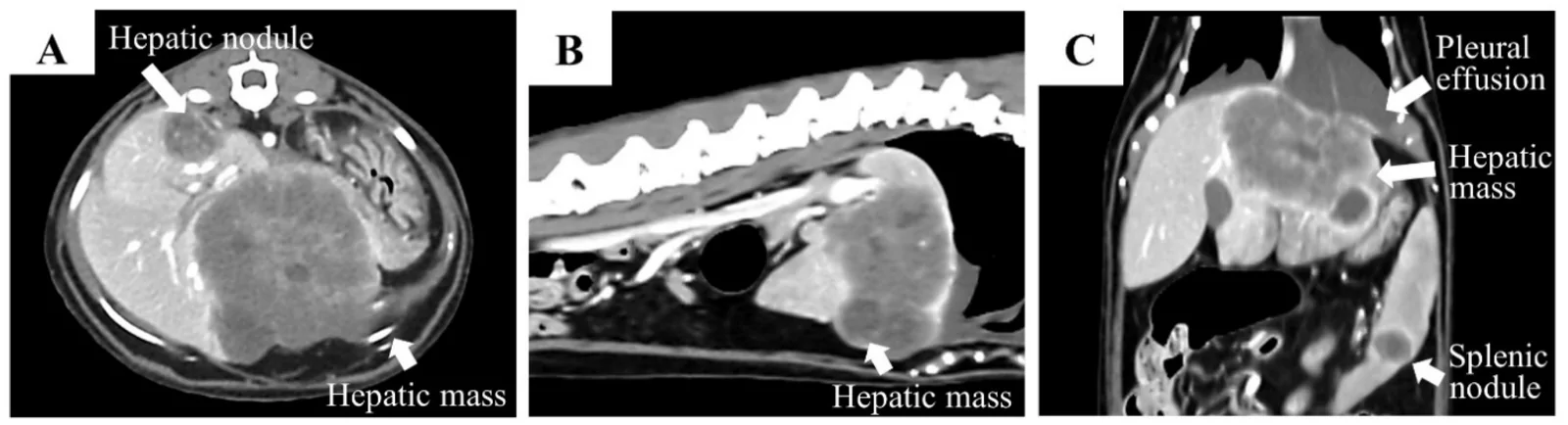
Computed tomography (CT) image of the thorax and abdomen from the affected cat. (**A**) A transverse plane CT image demonstrated a large multilobular cystic mass measuring 5.0 × 4.1 × 4.7 cm, with indistinct margins between the left medial, left lateral, and quadrate lobes. An additional smaller hepatic nodule was also identified. (**B**) A sagittal plane CT image demonstrated the same large multilobular cystic hepatic mass. No peritoneal effusion was detected. (**C**) A dorsal plane CT image revealed pleural effusion and a splenic nodule measuring 1.1 × 0.9 × 0.7 cm, which exhibited a contrast enhancement pattern similar to that of the hepatic mass on contrast-enhanced CT.

Computed tomography (CT; Optima CT660, GE HealthCare Japan Corporation, Tokyo, Japan) of the thorax demonstrated bronchovascular bundle thickening in the right middle and accessory lung lobes without evidence of a pulmonary nodule. Additionally, abdominal CT revealed a large multilobular cystic hepatic mass (Figure 2A,B). A hypodense splenic nodule (Figure 2C) and multiple pancreatic hypodense nodules were also identified. No peritoneal effusion was observed.

Under general anesthesia, an ultrasound-guided percutaneous core biopsy of the liver mass was performed using a 16-gauge semiautomatic SuperCore™ biopsy needle (Argon Medical Devices, Inc., Athens, TX, USA) for histopathologic evaluation, and a total of four tissue cores were collected and immediately fixed in 10% neutral-buffered formalin for 24 h. The specimens were routinely processed, embedded in paraffin, sectioned to 4–5 µm, and stained with hematoxylin and eosin. Ultrasound-guided fine-needle aspiration of the liver mass and cholecystocentesis were performed using a 22-gauge, 1.5-inch needle with a 10 mL syringe. The liver aspirate was submitted for cytologic examination, whereas the bile sample was submitted for cytologic examination and bacterial culture. The cat was fasted for 12 h prior to administration of propofol (8 mg/kg; Troypofol, Troikaa Pharmaceuticals Ltd., Gujarat, India). Hematologic, biochemical, and coagulation tests evaluated within 24 h prior to the procedures were within normal limits except for mild nonregenerative anemia (Table 1 and Table 2). No hemorrhagic or post-anesthetic complications were observed.

Liver cytology revealed clusters of oval-to-polygonal neoplastic cells consistent with carcinoma (Figure 1B). Histopathologic examination of the core liver biopsy subsequently revealed an infiltrative epithelial neoplasm composed of oval-to-polygonal neoplastic cells with moderate eosinophilic cytoplasm and large round-to-oval nuclei with marked anisokaryosis. The neoplastic cells were arranged in solid sheets and variably sized tubules supported by a thin fibrous stroma, suggesting biliary differentiation. These histologic features are consistent with a carcinoma of bile duct origin. The bile was dark green and non-purulent, with no cells or microorganisms on cytology, and both the aerobic and anaerobic cultures were negative.

We decided to employ palliative management with chest tube placement to improve quality of life 7 days after the definitive diagnosis. The medical therapy was mainly focused on supportive treatments and included hepatoprotective support (S-adenosyl-L-methionine, 20 mg/kg PO q24h, Samylin^®^ Small Breed, VetPlus Ltd., Lytham, UK; ursodeoxycholic acid, 23 mg/kg PO q24h; Ursolin^®^, Berlin Pharmaceutical Industry Co., Ltd., Bangkok, Thailand), pain control (gabapentin, 8 mg/kg PO q12h, Berontin^®^ 100, Berlin Pharmaceutical Industry Co., Ltd., Bangkok, Thailand), and iron supplementation (elemental iron, 5 mg/cat PO q24h, Ferro-B^®^, Intervetta Co., Ltd., Bangkok, Thailand). Administration of these medications was initiated at the initial visit to the referral hospital and continued throughout the clinical course.

An indwelling chest tube was surgically placed to facilitate intermittent pleural fluid drainage at home for 90 days. The owner monitored pleural fluid volume and appearance, respiratory signs, activity, and temperament at home, while the cat underwent weekly to monthly hospital re-evaluations including hematologic and biochemical testing and ultrasonographic assessment of hepatic mass size and adjacent tissue involvement. Approximately 30–40 mL of serosanguineous pleural effusion was drained per day. During the follow-up period, the cat developed azotemia 54 day after the definitive diagnosis, characterized by increased blood urea nitrogen and creatinine concentrations (106 mg/dL and 2.7 mg/dL, respectively), raising suspicion of a stage III acute kidney injury (AKI) as per the guidelines of the international renal interest society (IRIS). Subcutaneous fluid therapy was provided, and a subsequent reassessment was consistent with stage I chronic kidney disease (CKD) based on IRIS CKD staging criteria, with blood urea and creatinine concentrations of 60 mg/dL and 1.63 mg/dL, respectively. At the final visit, hematological analysis showed normocytic, normochromic, and regenerative anemia with leukocytosis (Table 1). Serum biochemical analysis revealed azotemia, mild hypoalbuminemia, and hyperfibrinogenemia (Table 2). Despite continued palliative medical management, the cat’s quality of life declined due to progressive neoplastic disease, and it was humanely euthanized, with the owner’s consent, 98 days after definitive diagnosis. Subsequently, a necropsy was performed to confirm the origin of the effusion.

**Figure 3:**
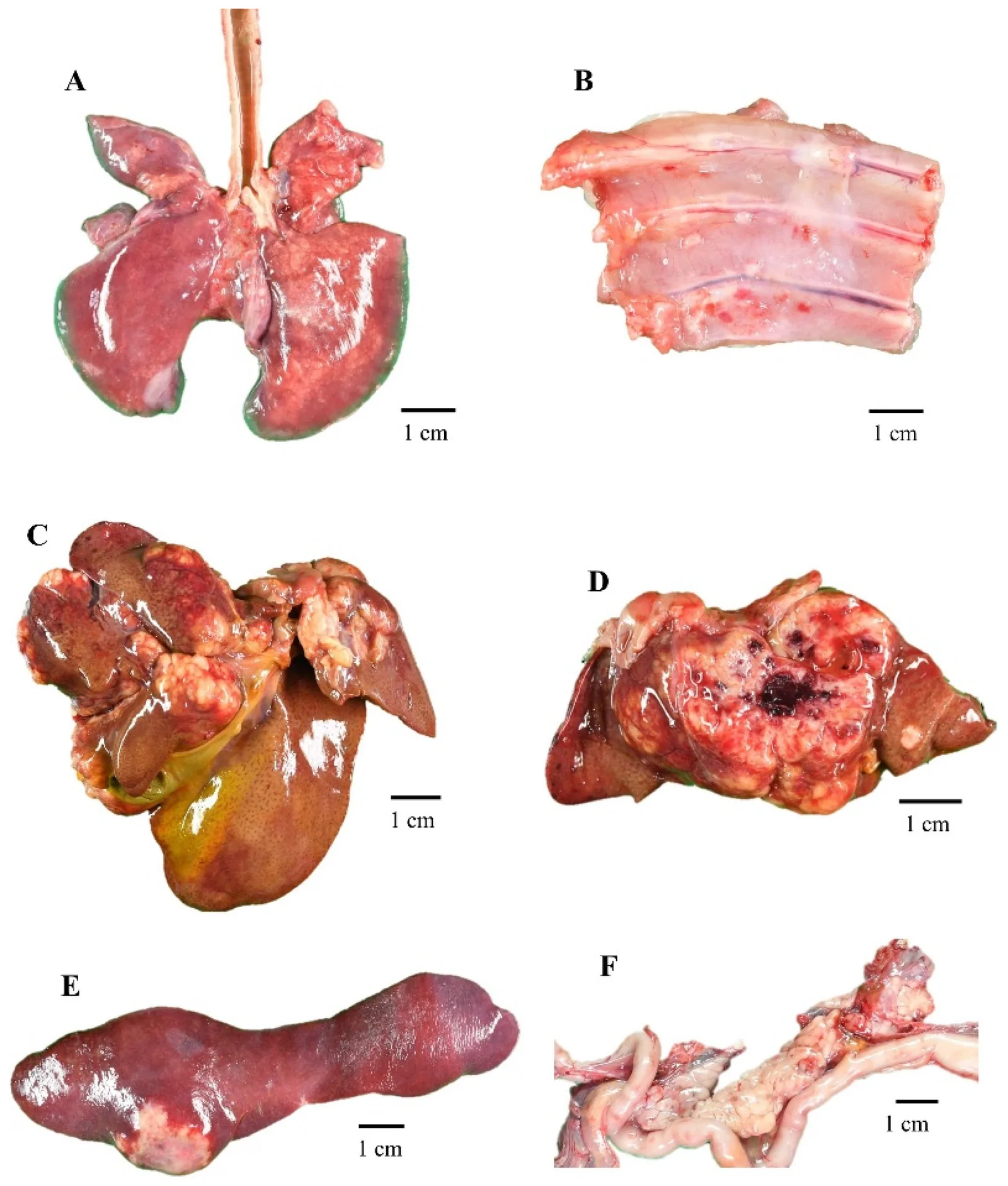
Gross evaluation during the necropsy of the affected cat. (**A**) The lungs were dark red and noncollapsed with mottled parenchyma. (**B**) The intercostal muscles contained multiple white nodules (3–5 mm). (**C**) The liver contained multiple masses. The largest (6 × 5 × 3 cm) involved the right medial, quadrate, left medial, and left lateral lobes with fibrous adhesions. (**D**) The cut surface of the hepatic mass was poorly defined and contained central necrosis. (**E**) The spleen had a nodule (2.5 × 1.5 cm) at the splenic head. (**F**) The pancreas was firm, and multiple nodules with irregular surfaces were identified.

The necropsy was performed 3 days after euthanasia, at which time approximately 50 mL of turbid red pleural effusion was found. No grossly visible pulmonary masses were identified in the lungs (Figure 3A), while multiple white nodules were observed in the intercostal muscles (Figure 3B). The abdominal cavity contained around 80 mL of turbid, dark red peritoneal effusion. Multiple hepatic masses (Figure 3C,D) and an irregular mass at the splenic head were identified (Figure 3E). The pancreas contained multiple nodules (Figure 3F), with additional small nodules present in the mesentery adjacent to the small intestine. An analysis of the postmortem pleural fluid revealed exfoliated neoplastic cells (specific gravity, 1.032; TNCC, 1797/µL; total protein, 36 g/L; fluid-to-serum total protein, ratio 0.51). The peritoneal fluid also included neoplastic cells (specific gravity, 1.023; TNCC, 5667/µL; total protein, 31 g/L; fluid-to-serum total protein ratio, 0.44).

**Figure 4:**
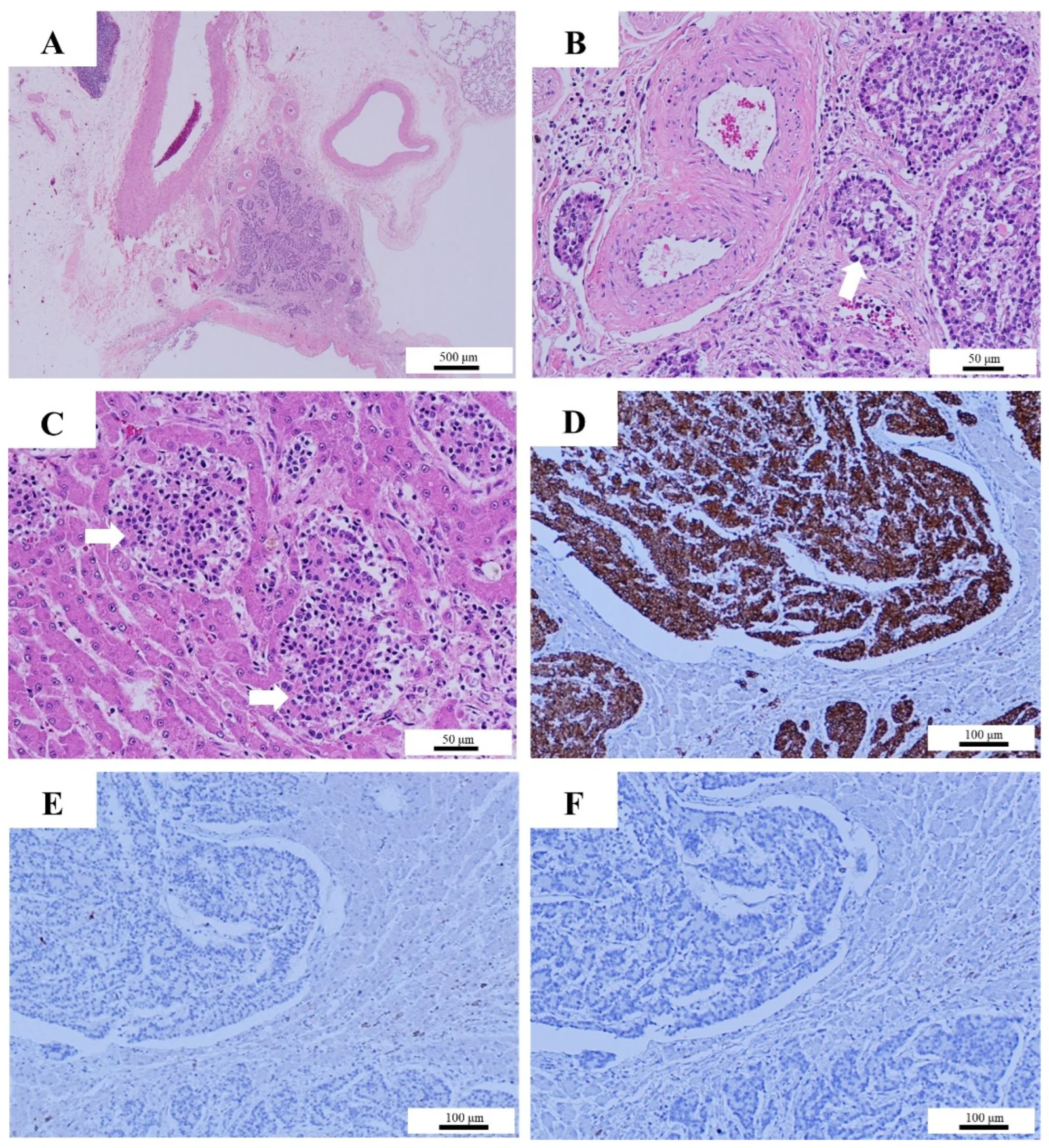
Histopathology and immunohistochemical staining of the affected cat were performed. (**A**) Hematoxylin-and-eosin staining of lung tissue, examined with a 4× objective demonstrated pulmonary parenchyma infiltrated by neoplastic cells that were arranged in glandular-to-poorly formed tubular structures. The neoplastic cells had pleomorphic vesicular nuclei and multiple prominent nucleoli. (**B**) Hematoxylin-and-eosin-stained lung tissue, examined with a 20× objective, showed neoplastic emboli within endothelial-lined lymphatic vessels (arrow). (**C**) Hematoxylin-and-eosin-stained liver tissue, examined with a 20× objective, showed multifocal areas of poorly demarcated, unencapsulated neoplastic infiltrates arranged in nests and islands (arrow). The neoplastic cells were polygonal, and the nuclei were highly pleomorphic with coarse chromatin and multiple prominent nucleoli. Hepatic neoplastic cells were subjected to immunohistochemical staining and examined with a 4× objective. (**D**) Immunohistochemical staining for pancytokeratin demonstrated strong diffuse cytoplasmic immunoreactivity for pancytokeratin, indicating epithelial differentiation. (**E**) Immunohistochemical staining for chromogranin A demonstrated no immunoreactivity. (**F**) Immunohistochemical staining for synaptophysin demonstrated no immunoreactivity.

Histopathology of the lung revealed pulmonary infiltration by clusters of polygonal epithelial neoplastic cells arranged in glandular-to-poorly formed tubular structures, consistent with metastatic carcinoma (Figure 4A). Neoplastic emboli were identified within the lymphatic vessels (Figure 4B) and arteriolar lumens. The visceral and parietal pleura showed no evidence of neoplastic infiltration. The hepatic mass demonstrated multifocal neoplastic cell infiltration (Figure 4C). Similar neoplastic infiltration was also observed in the spleen and intercostal muscles. The pancreas showed multifocal nodular proliferation of well-differentiated pancreatic acinar cells, a finding consistent with nodular hyperplasia.

Immunohistochemistry was performed on formalin-fixed, paraffin-embedded neoplastic tissues collected during the necropsy. All tissues including hepatic mass and metastatic lesions from the lungs, spleen, and intercostal muscles, were stained simultaneously with pancytokeratin, chromogranin A, and synaptophysin. Tissue sections were cut to 3–4 µm and mounted on positively charged glass slides. The sections were deparaffinized, rehydrated using ethanol, and subjected to heat-induced epitope retrieval. Ready-to-use primary antibodies against pancytokeratin (clone AE1/AE3; Leica Biosystems, Nussloch, Germany), chromogranin A (clone LK2H10; Cell Marque, Rocklin, CA, USA), and synaptophysin (clone 27G12; Leica Biosystems, Nussloch, Germany) were used. Antigen retrieval for pancytokeratin was performed in citrate buffer, pH 6.0, for 50 min. Chromogranin A antigen retrieval was performed in Tris–EDTA buffer, pH 9.0, for 60 min, whereas synaptophysin antigen retrieval was performed in citrate buffer, pH 6.0, for 60 min. The sections were incubated with the primary antibodies for 1 h at 37 °C. A previously confirmed squamous cell carcinoma was used as the positive control for pancytokeratin, whereas normal pancreatic tissue was used as the positive control for chromogranin A and synaptophysin. Immunoreactivity was detected using the Novolink Polymer Detection System (Leica Biosystems, Nussloch, Germany). Endogenous peroxidase activity and nonspecific protein binding were blocked using the reagents supplied with the kit. The sections were incubated sequentially with the Novolink Post Primary reagent and Novolink horseradish peroxidase polymer for 15 min each. Immunoreactivity was visualized using 3,3′-diaminobenzidine, and the sections were counterstained with hematoxylin. The liver mass showed strong cytoplasmic positivity for pancytokeratin (Figure 4D) and negative immunoreactivity for both chromogranin A (Figure 4E) and synaptophysin (Figure 4F), indicating epithelial differentiation and excluding a neuroendocrine origin. Neoplastic cells in the spleen, lungs, and intercostal muscles also showed cytoplasmic immunoreactivity for pancytokeratin and were negative for chromogranin A and synaptophysin. Based on the diagnostic imaging and histopathological findings during the necropsy and the absence of a primary carcinoma in the other organs examined, the cat’s state was considered most consistent with disseminated cholangiocarcinoma with histologically confirmed widespread metastases to the spleen, lungs, and intercostal muscles. The mesenteric nodules were grossly suspected to represent metastases, but they were not examined histologically.

## 3. Discussion

Feline cholangiocarcinoma typically presents with clinical signs related to the hepatobiliary system. However, in our case, the cat primarily had dyspnea due to recurrent pleural effusion, which was suspected to represent paramalignant pleural effusion and represented a manifestation distant from the primary tumor site.

Pleural effusion was classified based on cytologic findings, specific gravity, TNCC, total protein concentration, and the fluid-to-serum total protein ratio. A limitation of this report is that additional pleural fluid analyses, including analyses of glucose concentration and lactate dehydrogenase activity, were not performed. Although a decreased pleural fluid glucose concentration relative to that for serum glucose may indicate an infectious etiology [22], the omission of this parameter was unlikely to have affected the clinical diagnosis because both aerobic and anaerobic bacterial cultures yielded no bacterial growth. Although one of the fluid-to-serum protein ratios exceeded the protein threshold used in Light’s criteria (0.63), lactate dehydrogenase activity was unavailable, precluding complete application of Light’s criteria. Therefore, the pleural effusion was classified as a modified transudate according to conventional criteria based on total protein concentration, TNCC, and specific gravity. Nevertheless, Light’s criteria may improve classification of pleural effusions [23], particularly when fluid characteristics overlap, such as a low TNCC with high protein concentrations. Future studies of similar complex cases should include these parameters. Despite the minimal changes in pleural fluid characteristics throughout the clinical course, neoplastic cells were detected only in the postmortem sample, highlighting the value of repeated pleural fluid cytologic evaluation.

**Figure 5:**
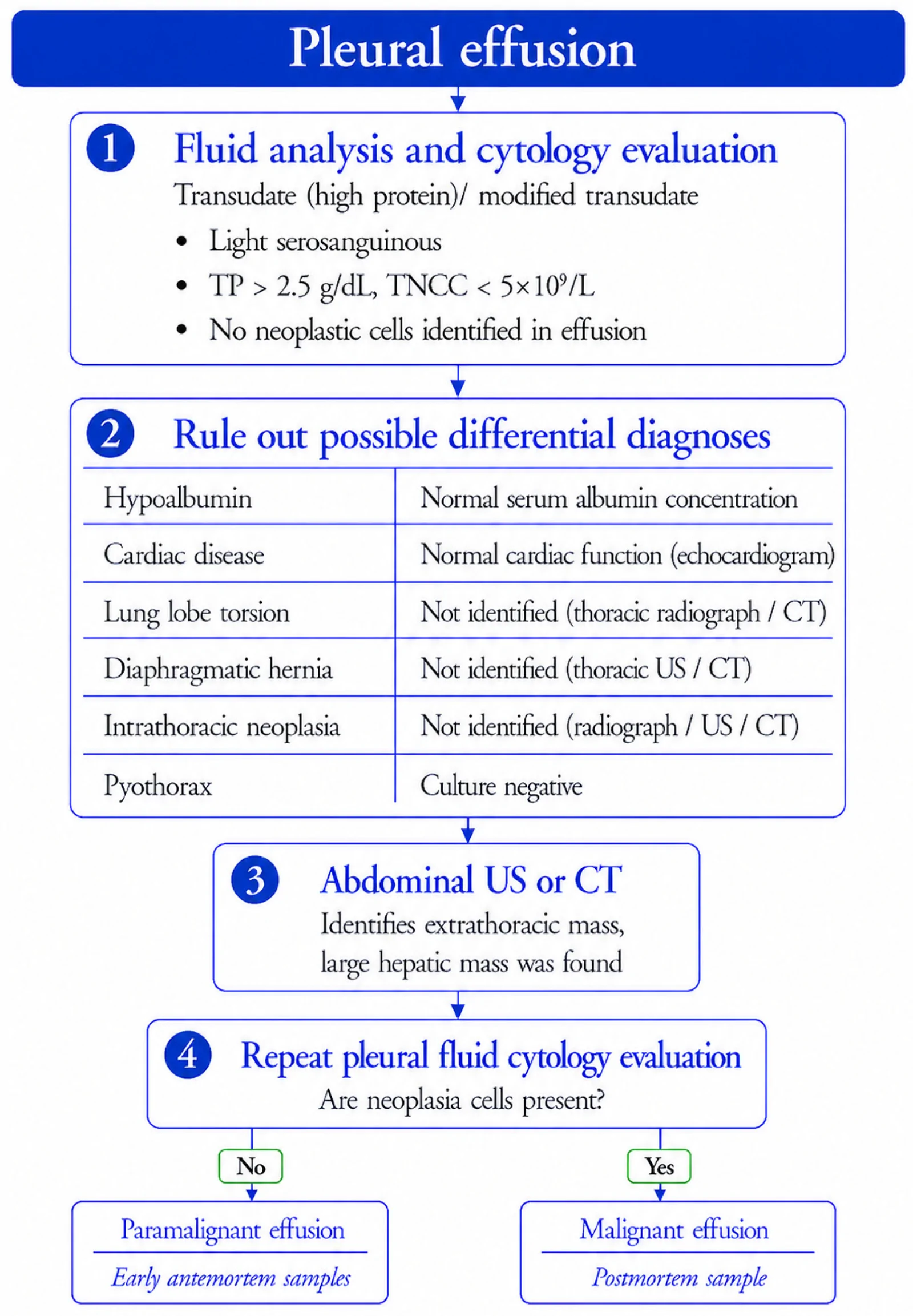
Diagnostic flowchart illustrating the challenges associated with diagnosing paramalignant pleural effusion in a cat with an extrathoracic mass. The initial fluid analysis revealed a modified transudate without neoplastic cells or a definitive cause. Following the exclusion of common differential diagnoses, abdominal ultrasonography and computed tomography revealed a large hepatic mass without intrathoracic lesions, raising suspicion of paramalignant pleural effusion. A postmortem cytological evaluation of pleural fluid during the necropsy revealed neoplastic cells. CT, computed tomography; TNCC, total nucleated cell count; TP, total protein; US, ultrasonography.

The pleural effusion raised suspicion of a paramalignant mechanism secondary to the hepatic mass after other causes were excluded, including infection, heart failure, thoracic masses or lung lobe torsion, and hypoalbuminemia (Figure 5). The absence of neoplastic infiltration in the visceral and parietal pleura in the necropsy, together with repeatedly negative antemortem pleural fluid cytology, supported our clinical interpretation that the patient was afflicted with suspected paramalignant pleural effusion. Therefore, the pleural effusion was potentially related to the neoplasm through indirect mechanisms, including impaired lymphatic drainage at parietal pleural stomata and increased pleural capillary permeability mediated by tumor-derived factors, such as vascular endothelial growth factor, without direct pleural invasion [3,7].

In addition, postmortem pleural fluid cytologic evaluation identified exfoliated neoplastic cells that were not detected in the two earlier antemortem pleural fluid samples. Similar findings have been reported in human patients with neoplasia-associated pleural effusion [4,5]. No additional pleural fluid cytologic evaluations were performed between diagnosis and the necropsy because the cat remained under palliative management, and the results were not expected to alter the therapeutic plan. Consequently, in this case, we cannot determine when neoplastic cells first became detectable. Nevertheless, the postmortem cytologic findings indicate that negative pleural fluid cytological results do not exclude metastatic malignancy, calling for repeated pleural fluid cytologic evaluation for cats with persistent or recurrent pleural effusion.

During the necropsy, the pleural effusion was determined to be associated with disseminated pulmonary metastatic carcinoma. Histopathological evaluation demonstrated pulmonary lymphovascular metastasis without evidence of visceral pleural invasion. In both human and feline medicine, hematogenous spread is the most common metastatic route [6,9,10]. In this case, histopathological examination revealed neoplastic emboli within pulmonary arteriolar lumens, indicating hematogenous dissemination. In addition, lymphangitic carcinomatosis was involved, as intralymphatic tumor emboli were identified. Pulmonary lymphangitic carcinomatosis accounts for approximately 6–8% of pulmonary metastases in humans [24] but remains poorly documented in feline patients. Our findings may support a mechanism in which neoplastic cells initially disseminate via the bloodstream and subsequently invade the interstitial lymphatics, resulting in infiltration of the pulmonary interstitium [25,26]. The absence of pulmonary nodules on CT did not exclude pulmonary metastasis. Histopathology confirmed pulmonary lymphovascular spreading, suggesting that the bronchovascular bundle thickening observed on CT represented early pulmonary lymphangitic metastasis despite the absence of discrete pulmonary nodules and the initially negative pleural fluid cytology. Bronchovascular bundle thickening has also been reported in dogs with metastatic carcinoma [10].

Widespread abdominal metastases were identified. Although imaging suggested that multiple pancreatic nodules indicated metastatic lesions, necropsy confirmed nodular hyperplasia was present, highlighting the limitations of imaging in differentiating benign from malignant pancreatic nodules. Ascites was not detected on CT or abdominal ultrasonography during the initial diagnostic evaluation. However, it was identified during the necropsy, suggesting disease progression. In cats with hepatobiliary malignancies, progressive tumor growth, vascular invasion, or compression of portal vasculature may serve to increased portal pressure and subsequent ascites formation, which may have occurred in this case.

Immunohistochemical staining for biliary epithelial markers (Cytokeratin 7 and Cytokeratin 19) and hepatocellular markers (Hepatocyte Paraffin 1 and arginase-1) was not performed because these antibodies were unavailable at our institution. Additionally, negative-control sections were not employed, representing a limitation of the immunohistochemical evaluation. The pulmonary, splenic, and intercostal lesions shared the same histomorphologic and immunohistochemical features with the hepatic mass, indicating metastatic spread from a common primary tumor, and no primary malignant epithelial neoplasms were identified in any of the other organs examined, supporting the notion that the liver was the most probable primary site. Therefore, although the dominant hepatic location, tubular architecture, fibrous stromal support, metastatic pattern, and pancytokeratin immunoreactivity were considered most consistent with cholangiocarcinoma, the diagnosis was based primarily on the histomorphologic features and the available immunohistochemical findings. The absence of additional lineage-specific immunohistochemical markers represents a limitation of this case report.

The cat described in this report survived 98 days after the definitive diagnosis, a period shorter than the reported median survival of 270 days (range: 0–889 days) for feline cholangiocarcinoma. This may be explained by the absence of a surgical intervention, as previous studies have demonstrated significantly shorter survival in nonsurgical cases relative to surgical cases [12], as well as the advanced stage of the disease with pulmonary involvement, which are associated with reduced survival times [19,20].

## 4. Conclusions

In this case report, we have presented a case of recurrent pleural effusion initially suspected to be paramalignant, causing dyspnea as a primary presenting sign of feline cholangiocarcinoma. Histopathological analysis confirmed that both lymphatic and hematogenous pulmonary metastatic spread may have contributed to fluid accumulation. Neoplasia-associated pleural effusion may vary over time, as malignant cells may fail to exfoliate into the pleural fluid. Such effusion may initially be considered paramalignant despite metastatic disease elsewhere, and neoplastic cells may not be detected until later pleural fluid samples are examined. Serial pleural fluid analysis and cytological evaluation are therefore recommended. Clinically, recurrent pleural effusion in cats may indicate underlying extrathoracic neoplasia as a potential source of effusion, even in the absence of neoplastic cells in pleural fluid or discrete pulmonary nodules on thoracic imaging. Pulmonary metastasis may occur without overt nodular lesions.

## Figures and Tables

**Table 1:** Hematological parameters of the affected cat at initial presentation and follow-up time points.

Parameter	First Visit	Day 0 (Definitive Diagnosis)	Day 98	Reference	Unit
Hemoglobin	9.5	10.7	7.3	10–15	g/dL
Packed cell volume	24.7	29.6	23.6	30–45	%
Red blood cells	6.11	7.17	4.99	5–10	×10^12^ cells/L
Mean corpuscular volume	40.4	41.3	47.3	39–55	fL
Mean corpuscular hemoglobin concentration	355	361	309	300–360	g/L
Absolute reticulocytes	12,220	31,548	133,732	0–50,000	/µL
Total white blood cells	10.22	7.18	30.75	5.50–19.00	×10^9^/L
Segmented neutrophils	8.42	5.25	29.21	2.50–12.50	×10^9^/L
Lymphocytes	1.28	0.97	0.31	1.50–7.00	×10^9^/L
Monocytes	0.28	0.19	0.92	0–0.85	×10^9^/L
Eosinophils	0.23	0.76	0.31	0–0.75	×10^9^/L
Basophils	0.01	0.01	0	Rare	×10^9^/L
Platelets	103	253	42	300–800	×10^9^/L
Plasma protein	76	60	68	60–75	g/L
Blood morphology	Platelet clumps	Platelet clumps	Polychromasia Giant platelets		
			Platelet clumps		

**Table 2:** Serum biochemical and coagulation profiles of the patient at initial presentation and follow-up time points.

Parameter	First Visit	Day 0 (Definitive Diagnosis)	Day 98	Reference	Unit
Sodium	148	N/A	N/A	147–162	mmol/L
Potassium	3.9	N/A	N/A	3.7–5.8	mmol/L
Calcium	10.2	N/A	N/A	8–11.8	mg/dL
Phosphorus	5.0	N/A	5.5	3.0–6.1	mg/dL
Blood urea nitrogen	30	30	88	19–34	mg/dL
Creatinine	1.53	1.53	1.65	0.9–2.2	mg/dL
Alanine aminotransferase	47	45	59	25–97	U/L
Alkaline phosphatase	30	32	29	0–62	U/L
Total bilirubin	0.5	N/A	N/A	0.1–0.6	mg/dL
Preprandial bile acids	<0.1	N/A	N/A	0–6.9	μmol/L
Ammonia	4	N/A	N/A	0–95	μmol/L
Glucose	150	120	112	60–130	mg/dL
Total protein	7.0	7.5	7.0	6.0–7.9	g/dL
Albumin	3.3	2.9	2.5	2.8–3.9	g/dL
Globulin	3.7	4.5	4.5	1.5–5.7	g/dL
Prothrombin time	8.4	9.1	8.8	6.5–11.5	s
Activated partial thromboplastin time	13.3	12.3	15.7	9.8–19.0	s
Fibrinogen	1.71	1.80	4.87	0.80–3.00	g/L

N/A, not available.

## Data Availability

The original contributions presented in this study are included in the article. Further inquiries can be directed to the corresponding author.

## Abbreviations

The following abbreviations are used in this manuscript:

AKI	Acute kidney injury
CKD	Chronic kidney disease
CT	Computed tomography
IRIS	International renal interest society
Kg	Kilogram
N/A	Not available
TNCC	Total nucleated cell count
